# Degradable Semi-Cycloaliphatic Epoxy Resin for Recyclable Carbon Fiber-Reinforced Composite Materials

**DOI:** 10.3390/polym17030293

**Published:** 2025-01-23

**Authors:** Kai Li, Zhonggang Wang

**Affiliations:** Department of Polymer Science and Materials, School of Chemical Engineering, Dalian University of Technology, Dalian 116024, China

**Keywords:** degradable, epoxy resins, carbon fibers, recyclable, composite materials

## Abstract

The development of an energy-saving method to recycle expensive carbon fibers (CFs) from end-of-life thermosetting resin-based CF-reinforced composites (CFRCs) is strongly desired because of the environmental and economic issues. The replacement of traditional thermosetting matrixes with controllably degradable epoxy resins provides a promising solution to this challenging task. In this work, a liquid acetal-containing semi-cycloaliphatic epoxy resin (H-ER) is designed and synthesized. After curing, H-ER shows simultaneously increased thermal stability, shearing strength, flexural strength, strain at break, and critical stress intensity factors by 126%, 26.5%, 17.0%, and 29.5%, respectively, in comparison with ERL-4221. Particularly, the cured H-ER is sufficiently resistant to organic solvents, bases, and weak acids but degrades rapidly in a modestly strong acidic aqueous solution, and the rate of degradation is controlled by modulating the acidity. GC-MS and FTIR spectra demonstrate that the degradation is indeed due to the cleavage of acetal linkages in the network, and the degradation-generated benzaldehyde may be reused as a raw material for the synthesis of the H-ER resin. More importantly, for the CFRCs using H-ER as a matrix, the CFs are readily recovered without detectable damage and are able to be recycled for CFRC fabrication.

## 1. Introduction

Epoxy resin-based carbon fiber-reinforced composites (CFRCs) are widely employed in many important industrial fields, such as aeronautics and astronautics, racing cars, sports apparatus, and transport tools [1,2,3,4,5,6], owing to their excellent thermal and mechanical stability and corrosion resistance. Currently, the epoxy resins used for CFRCs include bisphenol-based epoxy resins like diglycidyl ether of bisphenol-A epoxy resin (DGEBA), phenolic epoxy resins, and cycloaliphatic epoxy resins like 3, 4-epoxycyclohexylmethyl-3′, 4′-epoxycyclohexane carboxylate (ERL-4221). Nevertheless, the insolubility and infusibility nature of the thermosetting epoxy resins [7,8,9,10,11,12,13,14] results in touchy problems associated with the after-treatment of discarded CFRC products and the recovery of expensive carbon fibers [15,16,17,18,19].

A variety of methods for recycling waste CFRCs have been studied in the past years. In Schinner’s report, the composites were ground in a cutting mill, and the resulting grinds were reused as press-molding compounds to produce laminates [20]. Pickering and coworkers treated fiber-reinforced thermosetting composites through a fluidized bed combustion process at a bed temperature of 450 °C, and the fibers could be recovered [21]. Giorgini L recovered CFs by the thermal pyrolysis of CFRCs at a high temperature of up to 600 °C [22]. Although the CFs can be recovered by the above-mentioned processes, most of those obtained have been seriously damaged and lost their original performance. For instance, the mechanical property of regenerated fibers by the fluidized bed process was reduced by up to 50% [21].

In order to simplify the recovery process and reduce the energy cost, the fabrication of CFRCs with an epoxy resin matrix that is capable of chemical or thermal degradation at a lower temperature is favorable. In this way, the CFs are readily recovered under mild conditions and can maintain their original performance to a large extent. To this end, various epoxy resins with degradable functions were developed by introducing chemical or thermal labile bonds into the epoxy molecules such as phosphate, phosphite, sulfite, tertiary carbon-ether, and tertiary carbon-ether linkages [12,13,14,23,24,25,26,27,28].

Relative to thermal pyrolysis, the chemical degradation of epoxy resins is more energy-saving since it is carried out at a much lower temperature. Recently, Yu et al. developed a recyclable epoxy-based CFRP composite, where the thermosetting epoxy resin was able to dissolve in alcohol solvents by transesterification reactions at 160–180 °C. While further heating, the polymer solution could repolymerize to reform the thermoset [15,16]. Yamaguchi et al. prepared a cresol novolak-type epoxy resin containing degradable acetal linkages, from which the obtained carbon fiber-reinforced plastics could break down through the acidic treatment to recover carbon fibers [19]. Takahashi A et al. reported chemically degradable epoxy resins through the incorporation of disulfide linkages, which degraded into completely soluble fragments by base [11]. Shirai et al. developed degradable epoxies by means of the incorporation of sulfonate esters. The polymer networks became soluble at the elevated temperature of 120–200 °C [29]. Another approach is to introduce dynamic reversible covalent bonds, such as imine or Diels-Alder adducts, into the network that de-crosslinks at an elevated temperature [30,31,32]. It is worth pointing out that, to ensure sufficient service stability, the cured products of degradable epoxy resins are required to be well resistant to modestly high temperatures, organic solvents, and weak basic and acidic environments for a period of time. Meanwhile, the cured epoxy resins require rapid collapse under controlled conditions to allow the recovery of expensive carbon fibers from discarded CFRC materials.

Based on the above considerations, we synthesized a new semi-cycloaliphatic epoxy resin (H-ER) with one rigid phenyl and two epoxycyclohexyl groups linked through acetal bonds. An acetal bond is well known to be stable in the presence of bases and oxidizing and reducing agents but not in the presence of acids [33,34]. However, the sensibility of acetal to acid is tightly related to the steric hindrance and substituents, and the presence of large groups adjacent to the acetal bond is advantageous for its resistance to acids [35,36,37,38,39,40]. In this work, since the acetal bond in the H-ER molecule is encapsulated with bulky cycloaliphatic and phenyl groups, the cured H-ER network is therefore expected to possess sufficient stability in a weak, acidic environment but is hoped to be cleaved when the acidity of the solution reaches a certain degree. On the other hand, the introduction of a phenyl group in cycloaliphatic epoxy resins can improve thermal stability and mechanical properties, while cycloaliphatic epoxy resins usually display excellent processability due to their low viscosity prior to curing. [41,42]. These merits are especially favorable as matrixes for CFRCs. The synthesis and properties of acetal-containing cycloaliphatic H-ER and the derived H-ER/CFs composite materials, as well as the recovery and reusability of CFs, were studied in detail.

## 2. Materials and Methods

### 2.1. Materials

**Scheme 1:**
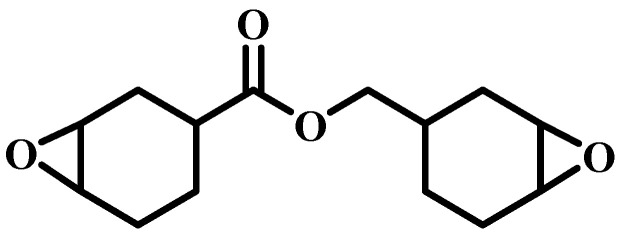
The chemical structure of ester-linked cycloaliphatic epoxy resin ERL-4221.

Cyclohex-3-enyl-1-methanol (CEM), 2KHSO_4_·KHSO_4_·K_2_SO_4_ (OXONE), acetic acid (ACA), oxalic acid (OXA), *p*-toluene sulfonic acid (*p*-TSA), anhydrous *n*-hexane (HEXA), dichloromethane (DCM), ethylenediaminetetraacetic acid (EDTA), and methanesulfonic acid (MSA) were from Shanghai Regent Company and used as received. Commercial epoxy resin ERL-4221 was obtained from Xinjin Chemicals Company (Wuhan, China) (Scheme 1). Hexahydro-4-methylphthalic anhydride (HMPA) and 2-ethyl-4-methyl-imidazole (EMI) were used as a curing agent and curing accelerator, respectively. Plain weave CF clothes (T300) were purchased from Jiangsu Hengshen Fiber Materials Company (Danyang, China). The chemical structure of ERL-4221 is depicted below:

### 2.2. Synthesis of 1-bis(3,4-epoxycyclohexylmethyl) Benzene (H-ER) and the Cured Product

CEM (42 g, 375 mmol), benzaldehyde (13.265 g, 125 mmol), *p*-TSA (2.375 g, 12.5 mmol), HEXA (187.5 mL), and 5 Å molecule sieve (25 g) were added into a 500 mL three-necked flask. The mixture was stirred at −8 °C for 6 h. The filtrate was firstly washed with 3 × 50 mL of aqueous solution of NaOH (15%) three times and then treated with 3 × 50 mL of deionized water three times. The organic phase was collected and dried over anhydrous MgSO_4_. After filtration, the solution was concentrated to remove the solvent. The crude product was distilled under vacuum and finally afforded 20.78 g of the intermediate product (H-OL) as a colorless liquid. Yield: 53%. FTIR (KBr): 3089 (s), 3022 (s), 2915 (s), 2836 (s), 1652 (s), 1494 (s), 1110 (s) 1051(s), and 1027 (s). ^1^H NMR (400 MHz, CDCl_3_, δ): 7.47 (d, J = 8 Hz, 2H, ArH), 7.36 (d, J = 8 Hz, 2H, ArH), 7.31 (d, J = 8 Hz, 2H, ArH), 5.67 (s, 4H, CH), and 5.54 (s, 1H, CH).

Subsequently, H-OL (9.37 g, 30 mmol), 18-crown-6 ether (0.9 g, 3 mmol), acetone (90 mL), and DCM (90 mL) were added in a 1000 mL flask. The mixture was reacted at −15 °C. Then, the solution of OXONE (45.0 g, 75 mmol) and EDTA acid (0.06 g, 0.2 mmol) was added, while the pH value was maintained between 7.4 and 7.9. The reaction mixture was stirred at −15 °C for 7 h. Then, the organic phase was washed with water and dried, which was concentrated; the crude product was distilled under vacuum to afford 8.85 g of H-ER as a colorless liquid. Yield: 86%. FTIR (KBr): 3091 (s), 2985 (s), 2923 (s), 2863 (s), 1494 (s), 1114 (s), 1045 (s), and 808 (s). ^1^H NMR (400 MHz, CDCl_3_, δ): 7.41(d, J = 8 Hz, 2H, ArH), 7.35(d, J = 4 Hz, 2H, ArH), 7.31 (d, J = 12 Hz, 1H, ArH), 5.46 (s, J = 6Hz, 1H, CH), and 3.23–3.29, (d, J = 8Hz, 4H, CH). ^13^C NMR (400 MHz, CDCl_3_, δ): 126–138 (Ar), 101 (–CH–O), 69 (–CH_2_–O), 51–52 (C–H on epoxide ring), 21–33 (cycloaliph, –CH_2_–, –CH–). ESI-MS *m*/*z*: [M + Na]^+^ calcd for C_21_H_28_O_4_Na, 367.4482; found, 367.1868.

The obtained H-ER and ERL-4221 were mixed, respectively, with HMPA and EMI at room temperature. The stoichiometric ratio of H-ER to HMPA was 1:0.95, while the added amount of EMI was 0.5 wt%. After being defoamed in vacuum condition, the mixture was cured at 100 °C for 2 h, 170 °C for 2 h, and 200 °C for 2 h.

### 2.3. Preparation of H-ER-Based CF-Reinforced Composite Materials

Six pieces of CF cloth with a dimension of 150 × 100 mm^2^ were pre-treated with acetone for 48 h and then dried at 80 °C for 6 h. The well-defoamed epoxy glue with the same composition as that described in the curing of epoxy resin was spread evenly on each layer of the fiber cloth, and the impregnated fiber clothes were heated in an oven at 50 °C for 6 h for pre-curing. Finally, the laminated carbon fiber clothes that were impregnated with pre-cured H-ER glue were transferred to a plate vulcanizer and pressed under 1 MPa at 100 °C for 2 h, 170 °C for 2 h, and 200 °C for 2 h.

### 2.4. Degradation of the Cured Epoxy Resin and CFRCs for the Recovery of Carbon Fiber Clothes

The cured H-ER was prepared into samples with the dimensions of 10 × 10 × 3 mm^3^. They were added into the solutions of 0.5 N MSA/THF/H_2_O, 0.5 N p-TSA/THF/H_2_O, 2.0 N ACA/THF/H_2_O, and 2.0 N OXA/THF/H_2_O, respectively. In all cases, the ratio of THF to H_2_O is 4:1 (*v*:*v*). At the refluxing temperature, the kinetic curves for degradation were obtained by recording the residual weight percentages of samples at different times.

The degradation of CFRCs and recovery of carbon fiber clothes were conducted as per the following procedure: The sample of the H-ER-based CFRC, with dimensions of 10 × 10 × 3 mm^3^, was placed into 50 mL of mixed solution of 0.5 N p-TSA in a THF/water (4/1 *v*/*v*) solvent. Then, the system was heated to a refluxing temperature, and degradation was carried out at this temperature for 6 h. Then, the regenerated carbon fiber cloth was washed with THF and acetone and dried under vacuum to a constant weight.

### 2.5. Instrumentation

Fourier-transform infrared spectra (FTIR) were performed on a Nicolet 5700 spectrometer (Burker Scientific Instruments, Billerica, MA, USA). The ^1^H NMR and ^13^C NMR spectra were recorded in CDCl_3_ on an INOVA-400 NMR spectrometer (Varian, Palo Alto, CA, USA). The mass spectrum was measured using an Agilent 6310 quadruple LC/MS (Agilent Technologies, Santa Clara, CA, USA). Gas chromatography–mass spectra were recorded on an Agilent 7000B triple quadrupole GC/MS (Agilent Technologies, Santa Clara, CA, USA). Dynamic viscoelasticity spectra were obtained on a TA DMA Q800 (TA Instruments, New Castle, DE, USA) at 2 °C min^−1^ with 1 Hz In N. Thermogravimetric curves (TGA) were recorded on a NETZSCH TG 209 thermal analyzer (NETZSCH Geraetebau GmbH, Bavaria, Germany) over a temperature range of 25–800 °C at 10 °C min^−1^ with a nitrogen gas flow at 60 mL min^−1^. Scanning electric microscopy (SEM) was conducted on a QUANTA 450 (Field Electron and Ion Co., Columbia, SC, USA) at a 20.0 kV accelerating voltage. All the samples were sprayed gold with a thickness of 0.5 mm. The fracture performance was measured in the same way as the three-point bending according to GB 4161-2007. Every group of five samples was measured at 25 °C, and their average values were calculated. The critical stress intensity factor (KIC) was calculated from the following equation: KIC = PCS/BW3/2·f (a/w), where PC is the maximum load obtained from the fracture load–deflection curve, S is the span width, B is the thickness of the specimen, W is the width of the specimen, a is the initial crack length, and f (a/w) is the geometric factor. Shearing strength tests were performed on the INSTRON-5567A (Instron Corporation, Norwood, MA, USA) according to the standard ISO 4587-1979.The flexural performance was tested on a universal testing machine (Instron 5565) with a loading rate of 2 mm/min on the sample rack with a span of 64 mm according to GB/T 9331-2008. The flexural strength (σ_f_), flexural modulus (E_f_), and strain at break (ε_f_) were calculated from the following equation: σ_f_ = 3FL/2bh^2^, E_f_ = L^3^k/4bh^3^ and εf = 6hs/L2.

## 3. Results and Discussion

### 3.1. Synthesis of Acetal-Containing Cyloaliphatic Epoxy Resin (H-ER)

**Scheme 2:**
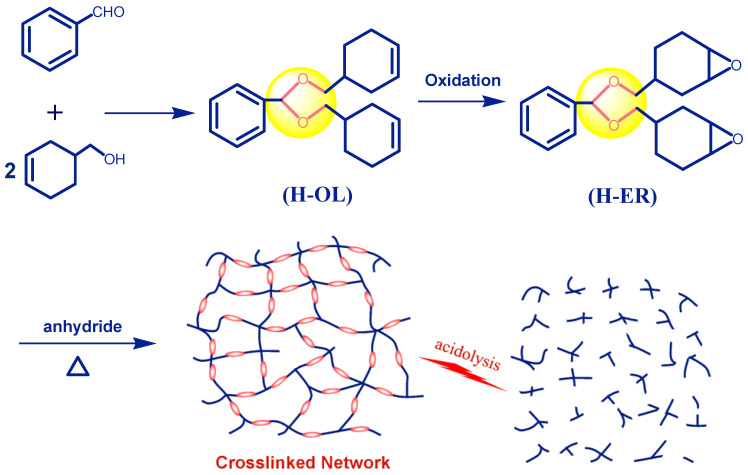
Synthetic routes to acetal-linked cycloaliphatic epoxy resin (H-ER), anhydride-cured network, and the depicted acidolysis process.

Using benzaldehyde and cyclohex-3-enyl-1-methanol as starting materials, the semi-cycloaliphatic diene precursor (H-OL) was prepared by acetalization reaction. Then, the epoxy resin H-ER with two epoxycyclohexyl groups linked with phenyl through acetal bonds was obtained through the oxidation reaction of H-OL with OXONE in the presence of the ethylenediamineretraacetic catalyst (Scheme 2). Compared with the furfural-based epoxy resin [43], the replacement of benzaldehyde with furfural can bring about two merits. Benzaldehyde is a cheaper and conveniently available raw material and, therefore, is more favorable from the viewpoint of future practical production and application. Moreover, the introduction of rigid phenyl groups into the network is expected to significantly improve the thermal and mechanical properties of the product, which are indeed demonstrated by the measured results, as discussed in Section 3.2.

**Figure 1:**
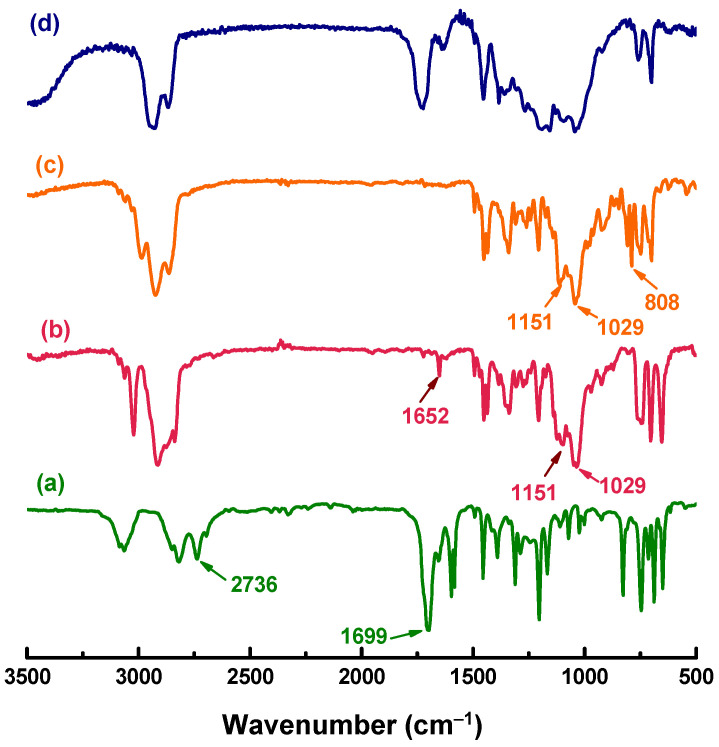
FTIR spectra of (**a**) benzaldehyde, (**b**) H-OL, (**c**) H-ER, and (**d**) cured H-ER.

**Figure 2:**
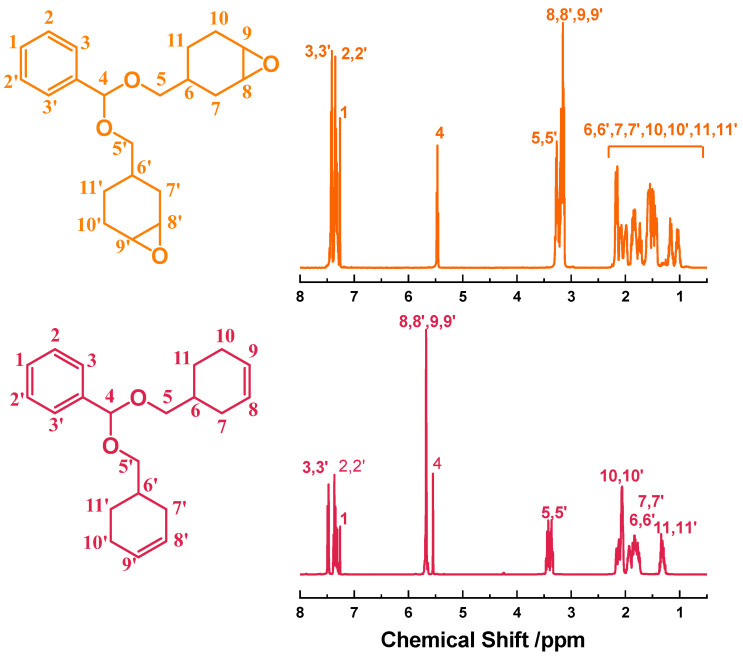
^1^H NMR spectra of H-ER and its olefin precursor H-OL.

In the FTIR spectra (Figure 1), the bands at 1029 and 1151 cm^−1^ are assigned to the acetal –C–O–C–O–C– in the diene precursor H-OL, whereas the absorptions at 1699 cm^−1^ and 2736 cm^−1^ corresponding to the aldehyde group of benzaldehyde disappear, demonstrating that the reaction between benzaldehyde and commercial cyclohex-3-enyl-1-methanol is complete. The newly emerged band at 808 cm^−1^ and the absence of the –C=C– band at 1652 cm^−1^ confirm that the –C=C– double bonds have been converted to epoxy groups. Moreover, after curing, the band of the epoxy group at 808 cm^−1^ disappears, indicating the complete curing reaction. In the ^1^H NMR spectra (Figure 2), the protons in the benzene ring are located at 7.47, 7.37, and 7.31 ppm, while the signals of –CH– and –CH_2_– in the cyclohexenyl rings are at 1.29–2.17 ppm. The protons in the epoxide rings appear at 3.12–3.19 ppm. In the ^13^C NMR spectra (Figure S1), the carbons on the benzene and epoxide rings are found at 126–138 ppm and 51–52 ppm, respectively. The other carbons of the cycloaliphatic CH or CH_2_ groups appear at 21–33 ppm. The ESI-MS spectrum (Figure S2) of the H-ER shows that the found molecular ion peak of M + Na^+^ (367.1868) agrees with the calculated value by the formula C_21_H_28_O_4_Na (367.4404 g/mol).

### 3.2. Thermal and Mechanical Properties of the Cured Epoxy Resin H-ER

**Figure 3:**
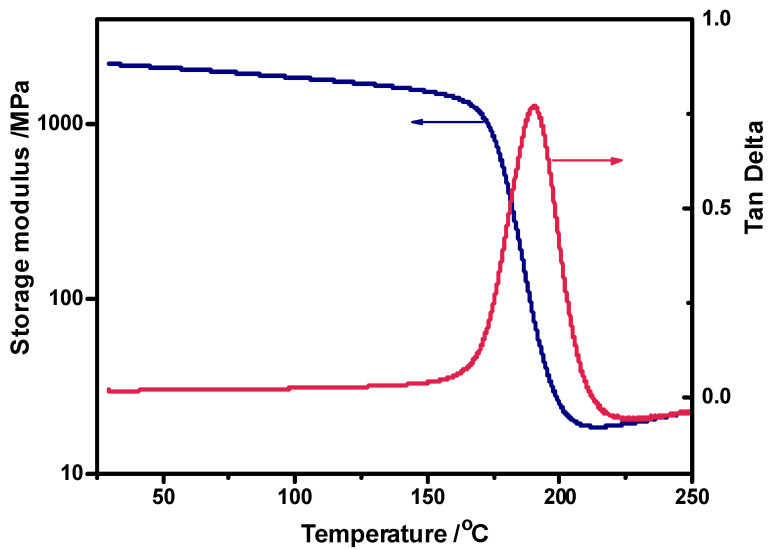
Dynamic viscoelasticity spectra of the anhydride-cured H-ER epoxy resin.

The semi-cycloaliphatic H-ER simultaneously contains an aromatic phenyl group and cycloaliphatic group, leading to its significantly superior thermal property compared to the wholly cycloaliphatic ERL-4221. Figure S3 shows the TGA trace of the cured H-ER measured in N_2_ at 10 °C/min. Its 2% weight-loss temperature is 324 °C, and the weight-loss at the maximum rate occurs at 378 °C, exceeding the values of the widely used commercial cycloaliphatic resin ERL-4221 (316 °C and 368 °C) [13], respectively. The excellent thermal stability ensures the durability of the H-ER-based CFRCs. In addition, the dynamic viscoelasticity of the cured H-ER was studied (Figure 3). Additionally, its crosslinking density (ρ) was estimated according to the storage modulus (E′) at T_g_ + 50 °C from the equation of ρ = E′/3RT [44], where R is the gas constant and T is the absolute temperature. The ρ value of the cured H-ER is 1.65 × 10^−3^ mol/cm^3^, much exceeding that of ERL-4221 (0.94 × 10^−3^ mol/cm^3^) [13], which can explain why the T_g_ value of H-ER (194 °C) is slightly higher than that of ERL-4221 (191 °C) [13], even though there are exit flexible C–O–C–O–C acetal linkages in the H-ER network. Moreover, the presence of a rigid benzene ring in the cured H-ER also results in an increased glass transition temperature in comparison with the furfural-based epoxy resin (186 °C) [43] measured under the same condition.

H-ER contains a rigid phenyl group, which belongs to semi-aliphatic epoxy resin. Moreover, the higher crosslinking density of H-ER also positively contributes to the mechanical toughness of the cured H-ER. As a result, the shearing strength of the cured H-ER is 7.45 MPa, which is remarkably superior to ERL-4221 (3.29 MPa) [14] and the furfural-based epoxy resin (4.70 MPa) [44], owing to the higher crosslinking density or the increased rigidity. Table 1 shows that the flexural modulus (E_f_) of the cured H-ER are comparable to those of ERL-4221, while the flexural strength (σ_f_), strain at break (ε_f_), and critical stress intensity factor (K_IC_) are 53.34 MPa, 7.42%, and 1.673 MN/M^3/2^, increased by 26.5%, 17.0%, and 29.5% compared to the values of ERL-4221, respectively. The excellent adhesion strength and mechanical toughness of H-ER are especially important as a resin matrix for CF-reinforced epoxy resin composites.

### 3.3. Degradation of the Cured Epoxy Resin H-ER

**Figure 4:**
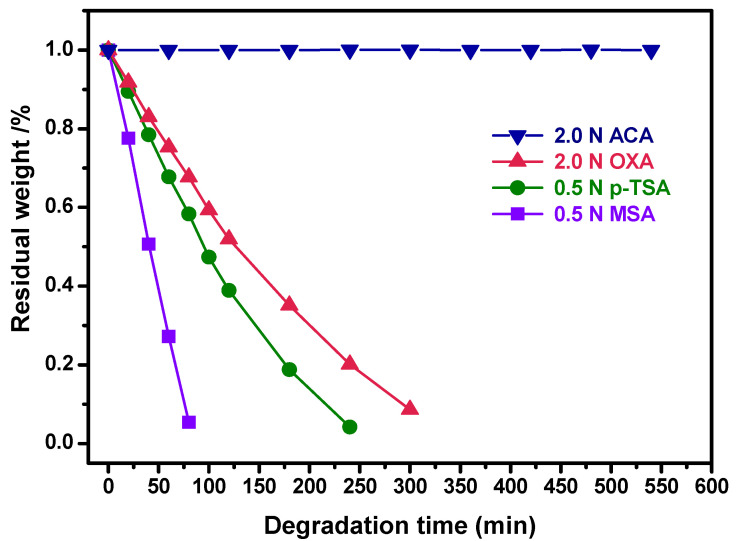
Degradation of the cured H-ER with time in different acidic aqueous solutions at the refluxing temperature.

The resultant cured H-ER is insoluble in organic solvents like acetone, chloroform, tetrahydrofuran, dimethyl sulfoxide, and N,N-dimethylformamide, as well as 1 N aqueous sodium hydroxide and sodium carbonate solution for 24 h at room temperature. Surprisingly, as shown in Figure 4, the sample did not exhibit degradation in the 2 N acetic acid/THF/H_2_O solution, even though it was treated at the refluxing temperature for 550 min, implying that the cured H-ER is sufficiently stable under harsh service conditions, such as being exposed to organic solvents or basic and weak acidic environments for a period of time.

Furthermore, the degradation of the cured H-ER was evaluated by treating the samples in the aqueous acidic solutions with enhanced acidity, such as oxalic acid (OXA), *p*-toluene sulfonic acid (*p*-TSA), and methanesulfonic acid (MSA). After changing the ACA with OXA, the sample rapidly degraded, and the degradation rate apparently accelerated with the increased acidity in the ranking sequence of MSA > *p*-TSA > OXA (Figure 4). For example, the cured H-ER lost about 73% of its weight after being immersed in the aqueous solution of MSA for only 60 min.

**Figure 5:**
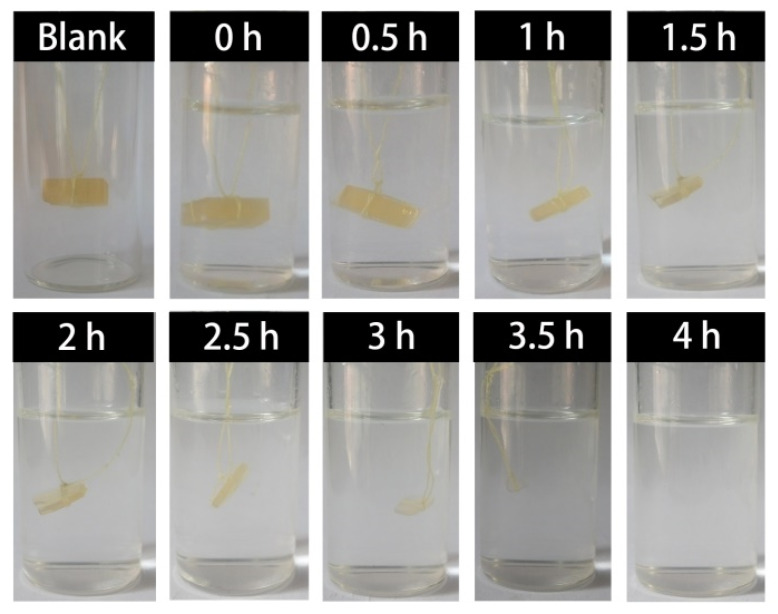
The observation of degradation with time for the cured H-ER treated with 0.5 N MSA aqueous solution.

The degradation of the cured H-ER was visibly observed through the changes in the sample in the 0.5 N MSA/THF/H_2_O solution, and the photographs are illustrated in Figure 5. The original sheet sample has the dimensions of 12 mm × 10 mm × 4 mm. As shown in Figure 5, the sample remarkably shrinks after the acid treatment and completely disappears after 4 h of acid treatment.

**Figure 6:**
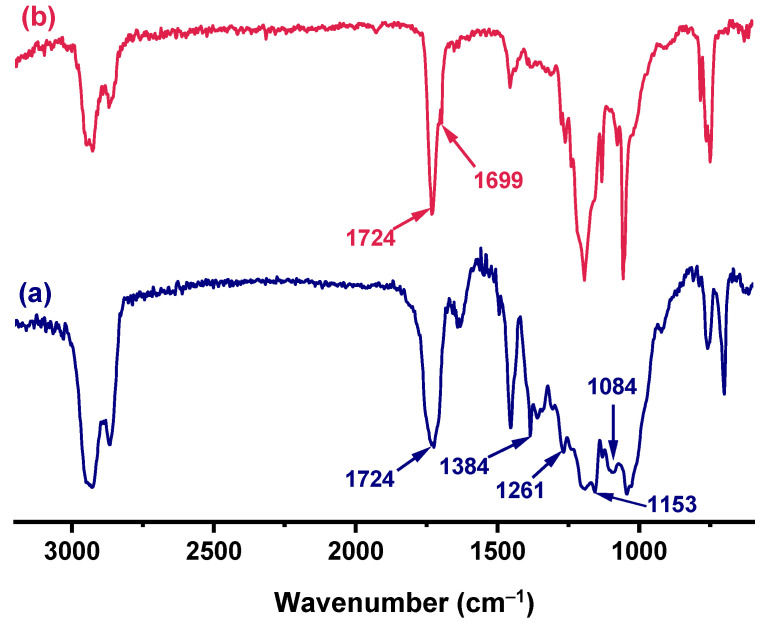
FTIR spectra of the cured H-ER (**a**) before degradation and (**b**) after degradation in MSA aqueous solution.

The degradation behavior was investigated by comparing the FTIR spectra of the cured H-ER before and after acid treatment. As shown in Figure 6, after acid treatment, the adsorption at 1724 cm^−1^ of the ester bond, derived from the curing of H-ER with hexahydro-4-methylphthalic anhydride, is almost unchanged. However, a shoulder peak at 1699 cm^−1^ appears, which is attributed to the stretching vibration of carbonyl in the aldehyde group. Moreover, the intensities of acetal linkage C–O–C–O–C at around 1010–1250 cm^−1^ apparently decrease, although its characteristic bands are overlapped with the C-O linkage of the ester group, demonstrating that acidolysis results from the cleavage of acetal rather than the ester linkages in the network.

**Figure 7:**
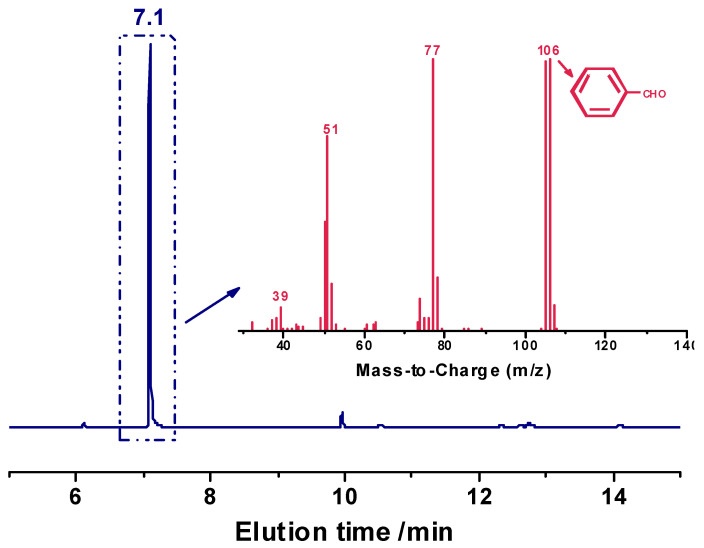
GC-MS spectra of the acidolyzed products of cured H-ER treated in MSA aqueous solution.

The degradation of the acetal-linked epoxy resin network was further examined by the analysis of the acid-degraded solution by GC/MS. Figure 7 displays that the strong peak at the elution time of 7 min in the GC spectrum is assigned to benzaldehyde since its molecular ion peak (106) is consistent with the molecular weight of benzaldehyde (106 g/mol), which also supports that the degradation of the cured epoxy resin is indeed due to the cleavage of acetal bonds. Moreover, the degradation-generated benzaldehyde may be reused as raw material for the synthesis of the H-ER resin.

### 3.4. Recycling of Carbon Fibers from the H-ER-Based CFRCs

**Figure 8:**
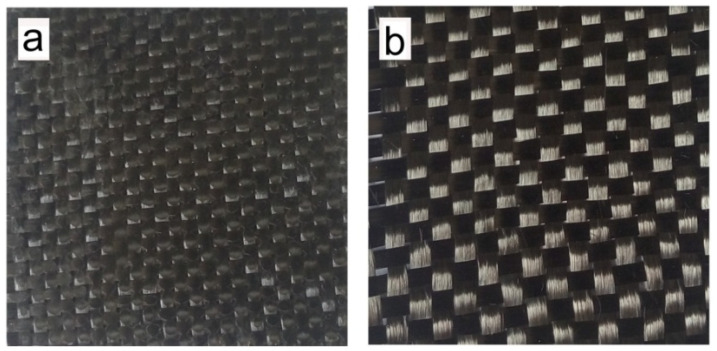
Photographs taken by digital camera of (**a**) H-ER-based laminated CFRCs without acid treatment and (**b**) the recovered carbon fiber cloth from 0.5 N MSA aqueous solution.

**Figure 9:**
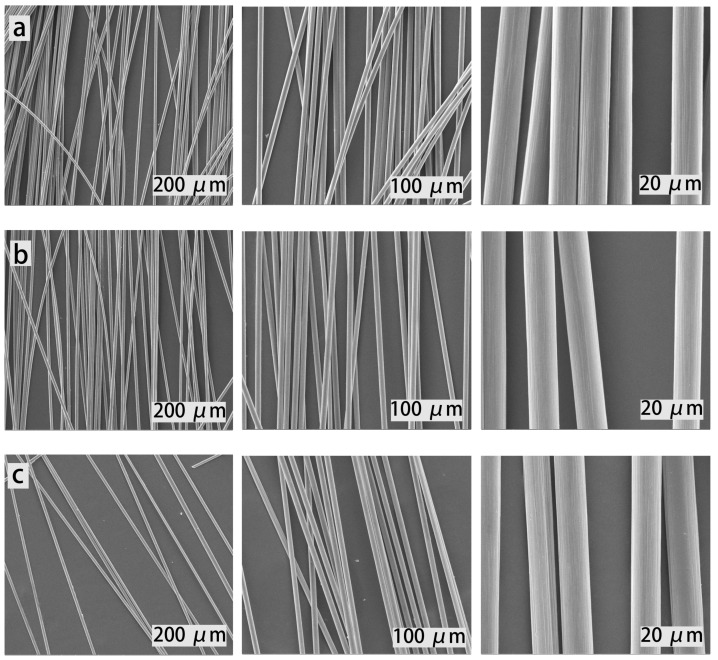

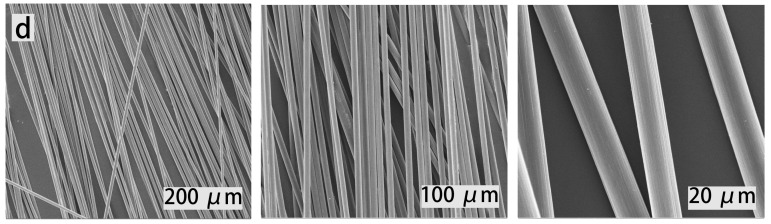
The SEM images of virgin and recycled CFs: (**a**) the virgin CFs, (**b**) the first recycled CFs, (**c**) the second recycled CFs, and (**d**) the third recycled CFs.

The regeneration of carbon fibers from the CFRCs was carried out by treating the composite with the 0.5 N MSA/THF/H_2_O solution at the refluxing temperature for 30 min. The laminated CFRCs were prepared from CF cloth and H-ER epoxy resin cured with anhydride. The sample size was 30 × 30 × 2 mm^3^. Figure 8 shows the photographs of CFRC samples with and without acidolysis. It is seen that the acid-treated CF cloth well maintains its original texture. Furthermore, the recovered CF cloth was recycled three times for CFRC fabrication. After acid treatment with the same procedure, the recovered carbon fibers were observed by scanning electron microscopy (SEM). As illustrated in Figure 9, the acid treatment can completely remove the epoxy resin matrix from the CFs, and the surfaces of the recovered fibers are almost the same as the original ones. No residue of epoxy resin or damage is found on the surface of the recovered carbon fibers, implying that the recycling of CFs from the CFRCs using degradable H-ER epoxy resin as a matrix is feasible.

## 4. Conclusions

In summary, a new semi-cycloaliphatic epoxy resin containing acetal linkages (H-ER) was synthesized. The cured product exhibited excellent thermal and mechanical properties. Its T_g_ and initial decomposition temperatures are 194 °C and 324 °C, while the shearing and flexural strengths are 7.45 and 53.34 MPa, respectively, which exceed or are comparable to the values of the commercial epoxy resin, ERL-4221. The cured H-ER is sufficiently stable in organic solvents and basic and weak acidic solutions, ensuring service reliability in the working environment. Meanwhile, at an elevated acidity, for instance, in aqueous methanesulfonic acid solution, the cured H-ER network can rapidly degrade due to the cleavage of acetal linkages, and the degradation rate is controllable by adjusting the acidity of the solution. This peculiar degradation property enables the recovery of carbon fibers from the composite fabricated with acetal-containing H-ER matrix resin. The results show that after acid treatment in an aqueous acidic solution, the carbon fiber cloths are completely recoverable, and the recovered carbon fiber cloths could well maintain their original texture without detectable damage on the surface. Moreover, the regenerated CFs can be repeatedly used for the preparation of composite materials. The facile preparation method, low cost of raw materials, and rapid degradation of the epoxy resin under controlled temperature for the undamaged recovery of carbon fibers endow the resultant H-ER with promising applications.

## Figures and Tables

**Table 1:** Thermal and mechanical properties of the cured epoxy resins.

	Thermal Property			Mechanical Property				
Sample	T_g_^a^ (^o^C)	T_2%_^b^ (^o^C)	T_max_^c^ (^o^C)	σ_s_^d^ (MPa)	σ_f_^e^ (MPa)	E_f_^f^ (GPa)	ε_f_^g^ (%)	K_IC_^h^ (MN/M^3/2^)
H-ER	194	324	379	7.45	53.34	0.74	7.42	1.67
ERL-4221	191	316	368	3.29	42.17	0.73	6.34	1.29

^a^ Glass transition temperature; ^b^ 2% weight-loss temperature; ^c^ temperature at maximum weight-loss rate; ^d^ shearing strength; ^e^ flexural strength; ^f^ flexural modulus; ^g^ strain at break; and ^h^ critical stress intensity factor.

## Data Availability

The original contributions presented in the study are included in the article/supplementary material, further inquiries can be directed to the corresponding author.

## Supplementary Materials

The supporting information can be downloaded at: https://www.mdpi.com/article/10.3390/polym17030293/s1. Figure S1: 13C NMR spectrum of H-ER; Figure S2: MS spectrum of H-ER; Figure S3: TGA and DTG thermograms of the anhydride-cured H-ER epoxy resin.
