# Supplementary material for: Degradable Semi-Cycloaliphatic Epoxy Resin for Recyclable Carbon Fiber-Reinforced Composite Materials
Source: Polymers (Basel). 2025 Jan 23;17(3):293. doi: 10.3390/polym17030293 (PMC11820351; doi:10.3390/polym17030293)
Supplement: Supplementary file 1 [file polymers-17-00293-s001.zip › polymers-3397621-supplementary.pdf]

Supporting Information

Degradable Semi-Cycloaliphatic Epoxy Resin for Recyclable Carbon Fiber-Reinforced Composite Materials

Kai Li and Zhonggang Wang *

Department of Polymer Science and Materials, School of Chemical Engineering, Dalian University of Technology, Dalian 116024, China

* Correspondence: zgwang@dlut.edu.cn

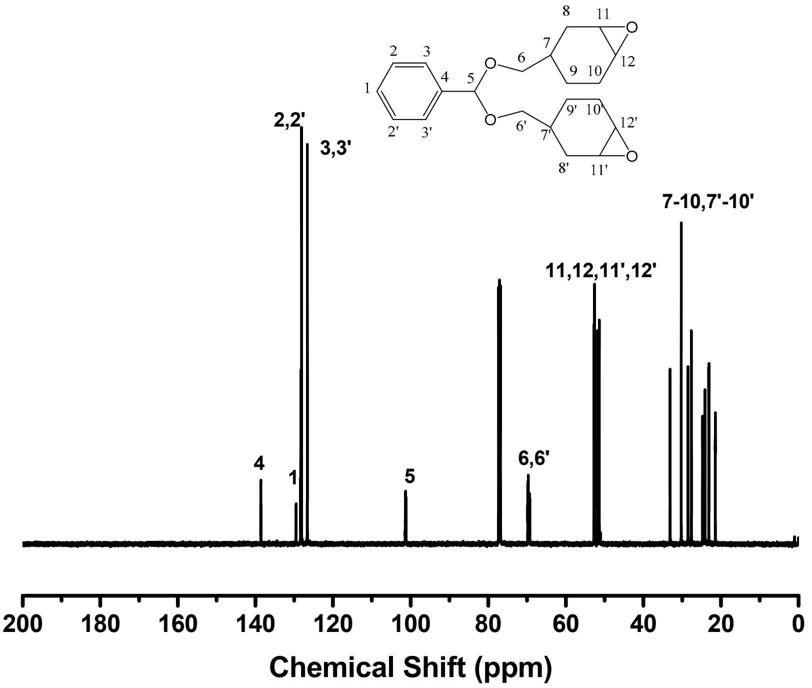

Figure S1. ^{13}C NMR spectrum of H-ER

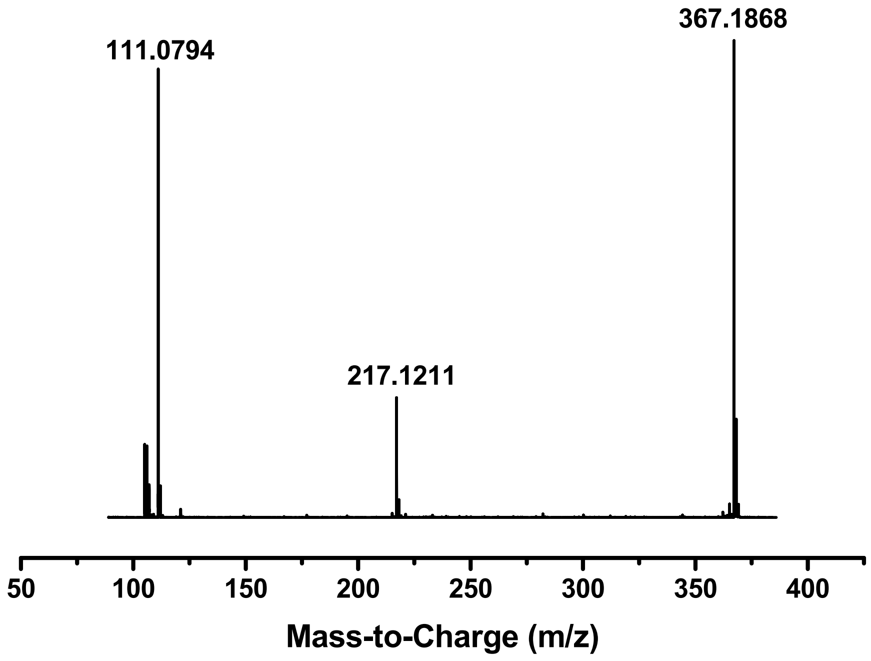

Figure S2. MS spectrum of H-ER

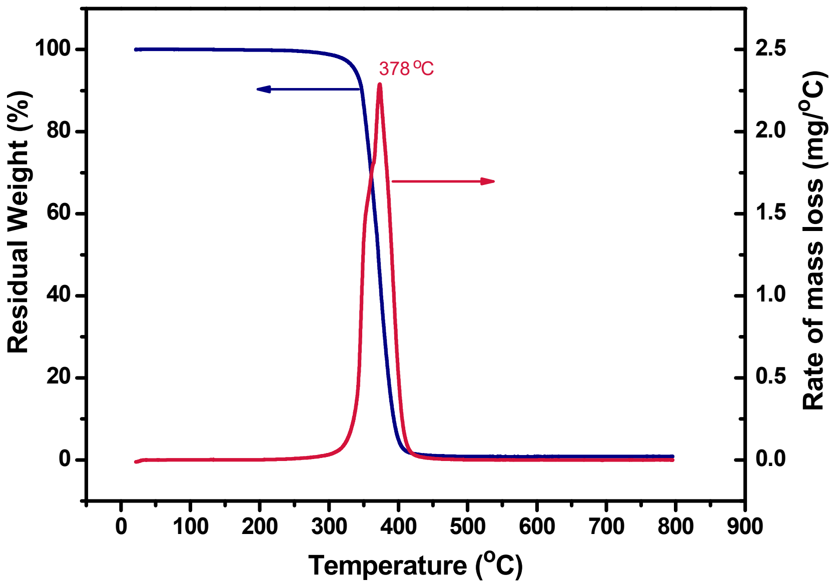

Figure S3. TGA and DTG thermograms of the anhydride-cured H-ER epoxy resin.
